# Evaluation of a Novel Virtual Reality Simulated Alternate Cover Test to Assess Strabismus: A Prospective, Masked Study

**DOI:** 10.1016/j.ajo.2024.08.042

**Published:** 2024-09-06

**Authors:** DIEGO MARTINEZ MORI, ASHIKA KUCHHANGI, JESSICA TAME, KAREN COOPER, LEILA HAJKAZEMSHIRAZI, MAANASA INDARAM, JEREMY D. KEENAN, JULIUS T. OATTS

**Affiliations:** University of California, San Francisco, California, USA; University of California, San Francisco, California, USA; University of California, San Francisco, California, USA; University of California, San Francisco, California, USA; University of California, San Francisco, California, USA; University of California, San Francisco, California, USA; University of California, San Francisco, California, USA; Francis I. Proctor Foundation, San Francisco, California, USA; University of California, San Francisco, California, USA

## Abstract

**• PURPOSE:**

A sensorimotor examination is the gold standard for strabismus diagnosis and quantification but requires a highly skilled examiner and may be limited by a child’s cooperation. Virtual reality (VR) employs eye-tracking technology to monitor eye position and may be able to measure strabismus. The aim of this study was to assess a prototype VR-simulated alternate cover test to detect and measure strabismus.

**• DESIGN:**

Prospective, masked diagnostic test study.

**• METHODS:**

Patients aged 5–18 years with visual acuity of 20/80 or better were prospectively enrolled to undergo strabismus measurements using a VR simulated alternate cover test (Olleyes, Inc., Summit, NJ) followed by an alternate cover test performed by a masked pediatric ophthalmologist or orthoptist. The main outcome measure was correlation between gold standard and VR-obtained strabismus measurements (in prism diopters [PD]) in primary gaze at near using Pearson correlation coefficients and Bland–Altman analysis with limits of agreement (LOA). A secondary measure was the diagnostic accuracy for the VR headset to detect strabismus.

**• RESULTS:**

A total of 85 participants were enrolled, mean ± standard deviation age was 10.8 ± 3.8 years, 45.9% (39/85) male. 40.0% (34/85) had strabismus: 17.7% (15/85) esotropia, 22.4% (19/85) exotropia, and 5.9% (5/85) vertical strabismus. 52.9% (18/34) of strabismus was intermittent. The overall correlation between VR and gold standard strabismus measurements was moderate but significant (r = 0.42, 95% CI 0.22, 0.58, *P* < .001), and correlation was strong for esotropia and constant deviations (r = 0.74, 95% CI 0.38, 0.91, *P* = .001 and r = 0.74, 95% CI 0.39, 0.91, *P* < .001, respectively). In participants with horizontal strabismus, Bland–Altman analysis showed a mean difference between standard and VR measurements of 3.55 ± 8.33 PD for esotropia (upper and lower LOA 19.89, −12.78 PD) and 17.15 ± 11.20 PD for exotropia (LOA 39.09 and −4.79 PD). Sensitivity for detecting strabismus was low: 27.6% (95% CI 12.7, 47.2), but specificity was high: 87.5% (95% CI 75.9, 94.8).

**• CONCLUSIONS:**

A prototype VR simulated alternate cover test showed a moderate but significant correlation with the gold standard sensorimotor examination and correlation was strong in those with esotropia and constant deviations. While the level of agreement demonstrated by this novel VR technology is promising, further improvements are needed before clinical deployment. However, this study demonstrates that VR has the potential to expand our ability to detect, measure, and monitor strabismus.

## INTRODUCTION

STRABISMUS, OR EYE MISALIGNMENT, IS ONE OF THE most common eye conditions in childhood, affecting 3%–5% of the population. Early detection and accurate diagnosis are crucial in the management of strabismus, as undetected strabismus can lead to amblyopia and irreversible vision loss. The current gold standard to diagnose and quantify strabismus is a sensorimotor examination which requires a specialist to perform direct, interactive measurements of eye alignment using prisms.^1^ The sensorimotor examination typically begins with the cover-uncover test, where an occluder is placed over one eye and then removed. If the nonoccluded eye moves to fixate, a tropia is present. Next, an alternate cover test is performed, which involves moving the occluder from one eye to the other with a pause between movements to allow fixation, detecting the presence of tropias and phorias. Finally, the alternate prism cover test uses prisms of increasing power over one eye until the deviation is neutralized, providing the magnitude of the deviation recorded in prism diopters. The sensorimotor examination has several limitations. First, access to this specialized care is limited by the number of pediatric ophthalmologists, with fewer than 1,100 pediatric ophthalmologists in the United States.^2^ Next, children may be fearful or uncooperative with testing, which can lead to inaccurate measurements.^3^ Last, variability in measurement techniques can lead to inter-observer variability and limit the generalizability of these measurements.^4–6^

To address these limitations, several emerging technologies have been evaluated to automate strabismus diagnosis and measurement. Studies have evaluated smartphone-based applications that simulate a corneal light reflex test using flash photography, measuring the location of the corneal light reflex, and applying an algorithm to detect and measure strabismus.^7,8^ Eye-tracking software has also been used to measure strabismus using near-infrared illumination to create corneal and pupillary reflection patterns which are recorded with a video camera or optical sensor. Using these measurements, a vector between the corneal light reflex and the pupil center is created and analyzed through an image processing algorithm to determine the position of the eye.^3,9^ This approach has also been used in combination with virtual reality (VR) technology in which infrared cameras embedded within VR headsets are used to measure the corneal light reflex and pupil center to determine the position of each eye.^10,11^

The Olleyes VisuALL ETS (Olleyes, Inc., Summit, NJ) is a VR platform that has previously been validated to perform perimetry in children and adults.^12^ Using eye-tracking software based on infrared-based eye position tracking using two arrays of six infrared sensors, this platform has a novel simulated alternate cover test where the VR device presents a stimulus to either eye and monitors eye position and movement.^13^ The goal of this study was to evaluate the performance of this novel VR-based simulated alternate cover test compared to the gold standard sensorimotor examination.

## METHODS

### • INCLUSION AND EXCLUSION CRITERIA:

This was a prospective, masked diagnostic test study approved by the University of California, San Francisco (UCSF) Institutional Review Board. This study conformed to the tenets of the Declaration of Helsinki and the Health Insurance Portability and Accountability Act. Written informed consent was obtained from parents and verbal assent from participants. Patients aged 5–18 years being evaluated in the UCSF Pediatric Ophthalmology were enrolled to undergo a sensorimotor examination performed as the standard of care followed by measurements using the VisuALL simulated alternate cover test. Exclusion criteria were best corrected visual acuity worse than 20/80 in either eye and children with nystagmus or physical or developmental impairments that limited their ability to follow commands or participate in either examination. Children that were unable to complete the VR calibration process were excluded.

### • SENSORIMOTOR AND CLINICAL EXAMINATION:

Participants underwent a sensorimotor examination performed by an orthoptist (JT, KC) or fellowship-trained pediatric eye provider (JO, LH, MI), prior to measurements with the VisuALL device. The sensorimotor examination was performed with or without optical correction as clinically indicated based on the type of strabismus. The examination included measurement of both horizontal and vertical components recorded in prism diopters (PD). Measurements were made in primary gaze at distance and near (20 feet and 13 inches, respectively). Clinical data including best corrected visual acuity (BCVA) and results of the sensorimotor examination (including presence, type, and magnitude of strabismus) were collected, and data were recorded using Research Electronic Data Capture (REDCap; Vanderbilt University, Nashville, TN). While examiners may have used both cover-uncover and alternate cover tests as clinically indicated, only data obtained from the alternate cover test at near was used for study and analysis purposes.

### • VR-BASED STRABISMUS ASSESSMENT:

Following the sensorimotor examination, participants underwent testing with the strabismus module using an the Olleyes VisuALL ETS VR headset. The operator was masked to the results of the sensorimotor examination. Measurements were obtained with or without optical correction as described above. The first portion of the examination is a tutorial where the examinee is given auditory and visual instructions. Next, a calibration stage ensures that the eye-tracking software can identify and follow the patient’s pupils. Then, the headset presents a simulated alternate cover test where a red stimulus is alternatively presented monocularly to each eye. During this, the position of each eye is tracked to detect and measure deviations that represent strabismus. Measurements are obtained in degrees and converted to prism diopters using the following formula: 100 * tan (angle in degrees) = angle in prism diopters.^14^ The device was calibrated daily and cleaned with 70% alcohol wipes before each examination.

### • POWER CALCULATION AND STATISTICAL ANALYSIS:

To achieve a desired intraclass correlation coefficient (ICC) of 0.8 with a precision of ±0.092, a minimum sample size of 60 participants was required.^15^ Correlation between strabismus measurements was assessed using Pearson correlation coefficients. In patients with strabismus, Bland–Altman plots were used to assess agreement and quantify the limits of agreement (LOA) between the magnitude of strabismus obtained from the sensorimotor examination and the VR platform. For Bland–Altman sub-analysis for patients with strabismus, strabismus was defined as a binary based on a manifest deviation in primary gaze at near of >5 PD for vertical deviations and >10 PD for horizontal deviations based on the gold standard alternate cover test as part of the sensorimotor examination. Strabismus measurements were obtained and recorded at the subject-level and for all analyses, the unit of analysis was per-subject (not per-eye). For our secondary measure assessing diagnostic accuracy, we calculated sensitivity and specificity of the VR module to detect strabismus based on the reference standard (index test) of the presence or absence of strabismus on the sensorimotor examination (using the same criteria as defined above). Analyses were performed using R version 4.3 (R Foundation for Statistical Computing) and *P* < .05 was considered statistically significant.

## RESULTS

### • DEMOGRAPHIC AND CLINICAL PATIENT DATA:

**FIGURE 1.**
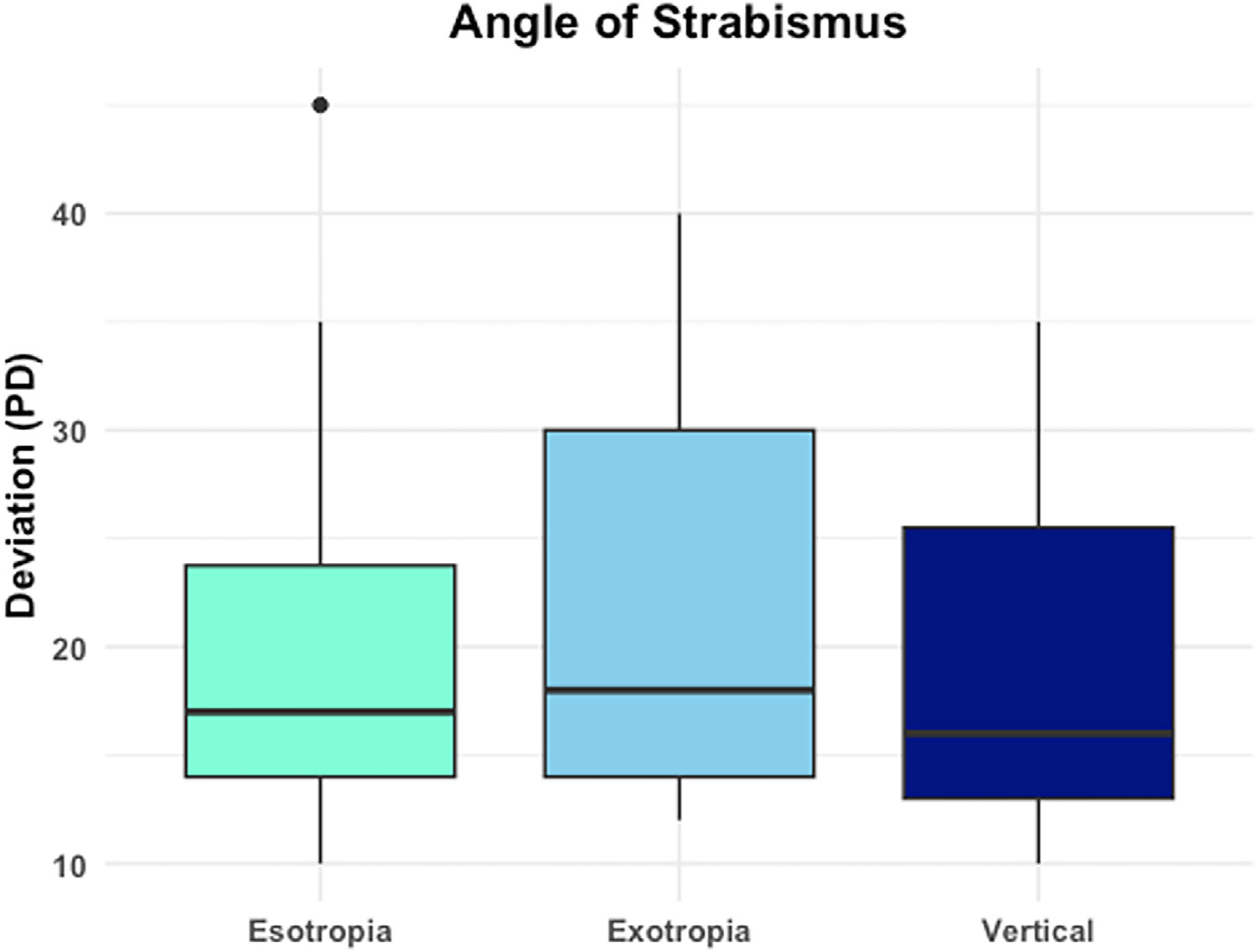
Boxplot demonstrating the magnitude of the strabismus angle among participants who had strabismus.

Of the 99 participants screened for the study, 14 were unable to complete the calibration, thus 85 participants were enrolled and included in the data analysis, mean ± standard deviation age was 10.8 ± 3.8 years, 45.9% (39/85) male. Of those enrolled, 40.0% (34/85) had strabismus: 17.7% (15/85) with esotropia, 22.4% (19/85) with exotropia, and 5.9% (5/85) with vertical strabismus. The majority of patients with strabismus had an intermittent deviation (52.9%, 18/34). Demographics are shows in Table 1. The mean strabismic angle was 16.33 ± 11.27 PD for esotropia, 22.42 ± 9.26 PD for exotropia, and 13.6 ± 13.05 PD for vertical strabismus (Figure 1). The majority of patients wore glasses (64%) and BCVA was 20/20 or better in 71%.

### • CORRELATION BETWEEN VR AND SENSORIMOTOR STRABISMUS MEASUREMENTS:

Among the entire cohort, there was a moderate correlation between strabismus measurements obtained with the VR device and the gold standard sensorimotor examination (moderate, r = 0.42, 95% CI 0.22, 0.58, *P* < .001; Table 2). Correlation was strongest in patients with esotropia or constant deviations (strong, r = 0.74, 95% CI 0.38, 0.91, *P* = .001 for esotropia and r = 0.74, 95% CI 0.39, 0.91, *P* < .001 for constant deviations). Correlation was weakest for participants with exotropia, intermittent, and vertical deviations (weak, r = 0.15, 95% CI −0.30, 0.56, *P* = .550; r = −0.28, 95% CI −0.66, 0.22, *P* = .265; r = −0.16, 95% CI −0.91, 0.84, *P* = .802, respectively).

### • BLAND–ALTMAN ANALYSIS:

**FIGURE 2.**
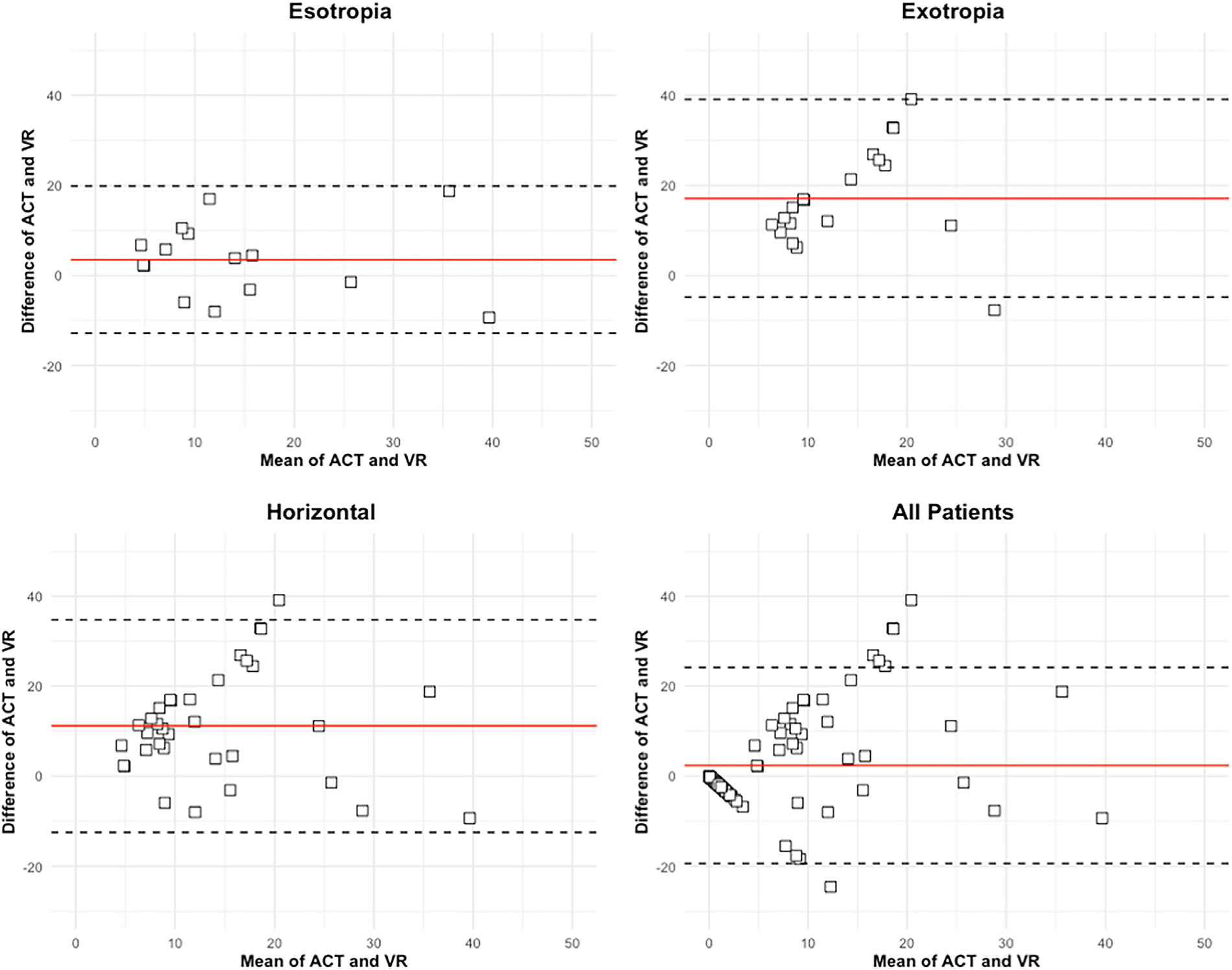
Bland–Altman plots with subgroup analysis based on the strabismus type. Each graph shows the difference between the virtual reality (VR) and alternate cover test (ACT) measurements on the y-axis and the mean on the x-axis. The central red line represents the mean difference and the dotted lines represent the lower and upper limits of agreement.

Bland–Altman plots are shown in Figure 2. For the entire cohort (*n* = 85), the mean difference between VR and ACT measurements was 2.40 ± 11.10 PD (upper and lower LOA −19.36 and −12.78 PD). In those with any type of strabismus (*n* = 34), mean difference was 11.15 ± 12.03 PD (upper and lower LOA 34.73 and −12.43 PD). For participants with esotropia (*n* = 15), the mean difference was smaller: 3.55 ± 8.33 (upper and lower LOA 19.89 and −12.78 PD). For those with exotropia (*n* = 19) and vertical strabismus (*n* = 5), the mean difference was larger (exotropia: 17.15 ± 11.20 PD, upper and lower LOA 39.09 and −4.79 PD; vertical: 10.88 ± 13.26 PD, upper and lower LOA 36.86, −15.11 PD). Bland–Altman analysis was not performed for the vertical strabismus subgroup due to the small number of patients. Generally, the VR underestimated the angle of the strabismus deviation and mean differences between VR and sensorimotor examination strabismus measurements are shown in Table 3. The largest difference was seen in participants with exotropia and the smallest in participants with esotropia.

### • DIAGNOSTIC ACCURACY OF VR FOR DETECTING STRABISMUS:

As a secondary measure, we evaluated the diagnostic accuracy of the VR to detect the presence or absence of strabismus using the applied binary cutoffs (>10 PD for horizontal strabismus and >5 PD for vertical based on the reference standard test). The overall sensitivity of the VR module for detecting strabismus was low: 27.6% (95% CI 12.7, 47.2), but specificity was high: 87.5% (95% CI 75.9, 94.8).

## DISCUSSION

In our study, we evaluated a novel VR-based module designed to detect and quantify strabismus using eye-tracking technology to evaluate eye position and alignment. Overall, measurements with this technology demonstrated a moderate correlation with the sensorimotor examination, though this correlation increased to strong in children with constant deviations and esotropia. On Bland–Altman analysis, the smallest difference between VR and reference standard measurements was seen with esotropia (3.55 PD) and the largest was with exotropia (17.15 PD). While the value for esotropia fell within a clinically acceptable margin (<5 prism diopters), the large limits of agreement and poor sensitivity demonstrate that further improvements are needed before deploying this module clinically.

While this is the first study to evaluate this VR-based simulated alternate cover test, several other studies have evaluated different VR modalities. A study evaluating a different VR platform, VIVE Pro Eye, similarly assessed the VR-measured strabismus angle compared to clinical examination and found high correlation (intraclass correlation coefficient 0.810–0.945).^10^ Similar to our study, they found that the VR platform tended to underestimate the magnitude of the deviation for participants with exotropia. There are several possible explanations for this discrepancy. First, accommodation may play a role. The simulated near target displayed in head-mounted displays may induce accommodation and convergence,^16^ which would lead to an underestimation of exotropic deviations. Additionally, many of the participants in our study with exotropia had intermittent deviations. Detecting an intermittent deviation requires a sufficient measurement latency to allow the eye to reach its final deviated position before re-gaining fixation. It is possible that the latency period of these devices requires adjustment or further study to optimize detection of intermittent exotropia.

Another study used the FOVE VR device in 17 patients with and without exotropia and found good correlation between VR- and doctor-obtained measurements.^11^ This group found a very small difference between measurement modalities in patients with exotropia (less than 0.7°), which may represent the importance of various VR parameters including specific pupil tracking technology, repeated measurements, and “stepwise approximation,” a process by which the targets presented to each eye are moved dynamically in response to eye position and movement.^11^ Eye-tracking technology has also been evaluated in non-VR settings such as wireless glasses used to measure corneal and pupillary reflections. This work demonstrated a strong correlation between automated measurements and those obtained by a physician (R = 0.9, <0.002) with a mean difference of −2.9 PD in their cohort of 69 children.^2^

Using VR to measure strabismus has several implications. First, this technology could be leveraged to screen patients in a clinic-based setting and triage which patients require examination by a specialist. Similarly, this could be used in a community-based setting as a screening tool similar to autorefractor devices.^17^ Given the low sensitivity observed in our study, further improvements in diagnostic performance would be required before using this technology as a screening platform. For patients with an established strabismus diagnosis, VR could allow for at-home measurements which could supplement telemedicine appointments or allow for asynchronous patient monitoring.^18^ Additionally, this technology could allow for decreased pre- or post-operative visits in patients requiring strabismus surgery. Several VR-based amblyopia therapies exist^19,20^ and it is feasible that future iterations could incorporate both diagnostic and therapeutic capabilities. While our data demonstrates that further refinements are needed to improve diagnostic accuracy, the possibilities to expand strabismus diagnosis are clear.

Our study has several limitations. First, our population was heterogenous with a wide range of types and magnitude of strabismus which may affect the accuracy of eye tracking technology. Similarly, given the age range of enrolled participants, anatomical differences relating to eye and head size may also impact this technology. Finally, diagnostic accuracy was a secondary outcome and the study was likely under-powered in this respect. Future studies can evaluate performance of this technology in adults with strabismus or sub-types of strabismus. Our study shows preliminary evidence that VR technology has the potential to expand the way in which we detect, measure, and monitor strabismus.

## Figures and Tables

**TABLE 1.** Participant Demographics and Clinical Information (*N* = 85)

Male sex, *n* (%)	39 (45.9%)

Age in years, mean (standard deviation)	10.8 ± 3.8
Measurements obtained in glasses, *n* (%)	55 (64.7%)
Manifest strabismus, *n* (%)	34 (40.0%)
Intermittent strabismus	18 (21.2%)
Esotropia	15 (17.7%)
Exotropia	19 (22.4%)
Vertical strabismus	5 (5.9%)

**TABLE 2.** Pearson Correlation Coefficients for VR and Sensorimotor Measurements

Subgroup	Correlation Coefficient (95% CI)	*P*-Value

All participants (*N* = 85)	0.42 (0.22, 0.58)	**< .001**
Esotropia (*n* = 15)	0.74 (0.38, 0.91)	**.001**
Exotropia (*n* = 19)	0.15 (−0.33, 0.56)	.550
Vertical strabismus (*n* = 5)	−0.16 (−0.91, 0.84)	.802
Intermittent strabismus (*n* = 18)	−0.28 (−0.66, 0.22)	.265
Constant strabismus (*n* = 16)	0.74 (0.39, 0.91)	**< .001**

**TABLE 3.** Bland–Altman Analysis: Difference Between VR and Sensorimotor Measurements

Type of Strabismus	Mean Difference,^a^ PD ± SD	Upper and Lower Limits of Agreement (LOA), PD

All participants (*N* = 85)	2.40 ± 11.10	−19.36, −12.78
Esotropia (*n* = 15)	3.55 ± 8.33	−12.78, 19.89
Exotropia (*n* = 19)	1715 ± 11.20	−4.79, 39.09
Vertical strabismus (*n* = 5)	10.88 ± 13.26	−15.11, 36.86
Intermittent strabismus (*n* = 18)	1700 ± 11.49	−5.53, 39.53
Constant strabismus (*n* = 16)	4.57 ± 9.03	−13.13, 22.27

a Primary near deviation as measured by alternate cover test minus virtual reality measurement.

PD, prism diopters; SD, standard deviation.
